# Psychosocial well-being index and sick leave in the workplace: a structural equation modeling of Wittyfit data

**DOI:** 10.3389/fpsyg.2025.1385708

**Published:** 2025-01-24

**Authors:** Rémi Colin-Chevalier, Bruno Pereira, Samuel Dewavrin, Thomas Cornet, Julien Steven Baker, Frédéric Dutheil

**Affiliations:** ^1^CNRS, LaPSCo, Physiological and Psychosocial Stress, Occupational and Environmental Medicine, CHU Clermont-Ferrand, Université Clermont Auvergne, Clermont-Ferrand, France; ^2^Biostatistics Unit, DRCI, CHU Clermont-Ferrand, Clermont-Ferrand, France; ^3^Cegid, Lyon, France; ^4^Centre for Health and Exercise Science Research, Hong Kong Baptist University, Kowloon Tong, Hong Kong SAR, China

**Keywords:** psychosocial factors, sick leave, structural equation modeling, psychosocial well-being, Wittyfit

## Abstract

**Background:**

Psychosocial well-being, which assesses emotional, psychological, social, and collective well-being, could help measure risk and duration of sick leave in workers.

**Objective:**

This study aims to build a structural equation model of a psychosocial well-being index based on 10 psychosocial factors and investigate its association with sick leave.

**Methods:**

Data of workers using Wittyfit was collected in 2018. Psychosocial factors (job satisfaction, atmosphere, recognition, work-life balance, meaning, work organization, values, workload, autonomy, and stress) were self-assessed using health-related surveys, while sick leave records were provided by volunteer companies.

**Results:**

A total of 1,399 workers were included in the study (mean age: 39.4 ± 9.4, mean seniority: 9.2 ± 7.7, 49.8% of women, 12.0% managers). The prevalence of absenteeism was 34.5%, with an average of 8.48 ± 28.7 days of sick leave per worker. Structural equation modeling facilitated computation of workers’ psychosocial well-being index (AIC: 123,016.2, BIC: 123,231.2, RMSEA: 0.03). All factors, except workload (*p* = 0.9), were influential, with meaning (*β* = 0.72, 95% CI 0.69–0.74), values (0.69, 0.67–0.70) and job satisfaction (0.64, 0.61–0.66) being the main drivers (*p* < 0.001). Overall, psychosocial well-being was found to be a protective factor for sick leave, with a 2% decreased risk (OR = 0.98, 95% CI 0.98–0.99, *p* < 0.001) and duration (IRR = 0.98, 95% CI 0.97–0.99, *p* < 0.001) per psychosocial well-being index point.

**Conclusion:**

The psychosocial well-being index provides a measure of psychosocial well-being and helps predict sick leave in the workplace. This new indicator could be used to analyze the association between psychosocial well-being and other health outcomes.

**Clinical trial registration:**

Clinicaltrials.gov, identifier NCT02596737.

## Introduction

Psychosocial well-being is a multidimensional concept that encompasses emotional, psychological, social and collective well-being (Larson, 1996), which relates to the concept of “quality of life” (Eiroa-Orosa, 2020). Conversely, psychosocial factors are workplace factors that can affect psychological and social well-being of employees, namely their psychosocial well-being, and potentially harm their physical and mental health (Thomas et al., 2020). Over time, the focus shifted toward understanding the psychosocial aspects of work, as in the Karasek model of the relationship between work demands/workload and work control/autonomy (Lu et al., 2019), or in Siegrists model of effort/reward imbalance. Further studies have even highlighted the link between the dimensions of these two models and life expectancy (beyond well-being) (Kivimäki et al., 2018). The emergence of occupational health psychology in the late 20th century further emphasized the importance of balancing work demands with factors like job satisfaction (Bagheri Hosseinabadi et al., 2018), psychological and social support (Penconek et al., 2021), recognition and rewards (AbuAlRub and Al-Zaru, 2008), work-life balance (Greenhaus et al., 2003), meaning of work, work environment (Ojala et al., 2018), organizational culture (Lu et al., 2019), and event stress (Steptoe and Kivimäki, 2012). Today, research continues to explore the dynamic interplay between these factors and their impact on both individuals and organizations.

Sick leave refers to a time period during which a worker is absent from work due to illness and granted a leave of absence. The phenomenon of sick leave is multifaceted and influenced by a variety of personal, social, and demographic factors, as well as organizational factors such as work-related factors both within and outside of the work environment. Such work-related factors include, non-exhaustively, poor work-life balance (Antai et al., 2015), poor psychosocial work environment (Catalina-Romero et al., 2015), low social support (Undén, 1996; Silva-Junior and Fischer, 2015), job dissatisfaction (Lardon et al., 2018; Tadesse et al., 2015), job stress (Tadesse et al., 2015), and low job control (Gimeno et al., 2004; Kottwitz et al., 2018). In addition to having a significant impact on the financial health and productivity of an organization (Asay et al., 2016; Nagata et al., 2018), sick leave appears above all to be a considerable risk for the health of workers (Johnston et al., 2019; Antczak and Miszczyńska, 2021).

Psychosocial well-being is an inclusive and complex concept and seems relevant for investigation via its multiple dimensions before assessing its putative effect on sick leave in the workplace. Yet, psychological well-being is a multidimensional construct (Hernandez et al., 2018), and exploring the relationships between multiple factors appears more relevant than focusing on a single explicative factor. Models for assessing psychosocial well-being, such as the Ryff model (Ryff and Keyes, 1995), the Psychological General Well-Being Index (Lundgren-Nilsson et al., 2013), and the Psychological Well-Being Scale network model (Blasco-Belled and Alsinet, 2022), are based on the assessment of combined psychosocial factors. These models are characterized by their multifactorial structure, since they examine several dimensions of psychosocial well-being, and by the fact that they are based on questionnaires containing a large number of interconnected items within the dimensions. A more condensed model could offer significant clinical benefits. Thus, the main objective of this study was to build a structural equation model of a psychosocial well-being index (PWI) which embraces multiple psychosocial factors: job satisfaction, atmosphere, recognition, work-life balance, meaning, work organization, values, workload, autonomy, and stress. A secondary objective was to measure the influence of the PWI on both risk and duration of sick leave.

## Materials and methods

### Recruitment

The software Wittyfit was created by Cegid (Lyon, France) in collaboration with the university hospital of Clermont-Ferrand. It is a web-based application used by many companies across different industries in France (Dutheil et al., 2017). Wittyfit allows companies to evaluate the well-being and psychosocial risks of their employees by gathering data on their feelings, with the goal of enhancing overall performance. Participation is voluntary, and the software provides a way for users to express themselves anonymously by responding to a variety of health-related surveys, which can help assess their general health status. The software then provides personalized feedback based on this information. Data collection for this study was limited to the year 2018. The study included all employees who had completed surveys using Wittyfit at least once during the year, whether they had completed fully or only partially completed the surveys. Sick leave data for 2018 was provided by two clients of Wittyfit. Before participating in the study, all subjects gave their informed consent, as outlined in the terms and conditions of Wittyfit. The study itself was approved by two regulatory bodies: the French data protection authority (CNIL), as well as the Ethics Committee of Clermont-Ferrand (South-East IV).

### Measures

#### Psychosocial factors

Psychosocial factors include job satisfaction, atmosphere, recognition, work-life balance, meaning, work organization, values, workload, autonomy, and stress. To evaluate all factors, Wittyfit uses non-graduated visual analog scales, which are relative and unique for each indicator, with a score range of 0–100. In other words, all 10 factors are measured using a single visual analog of the same name. For all factors except stress, a score of zero indicates a poor score in that factor, while a score of 100 indicates a good score. For stress, the lower the score, the better the worker’s feeling. The use of visual analog scales is common to measure health indicators, such as stress for example (Lesage et al., 2012; Dutheil et al., 2013; Dutheil et al., 2017). Workers were able to estimate their feelings related to each factor multiple times throughout the year. The average score for each factor was retained. If a worker expressed no feeling for a factor, we considered it a missing value.

#### Sick leave risk and duration

If a worker was absent due to illness during the year, his or her period of unavailability was measured in days. The total duration of sick leave was then determined by adding up all the periods of unavailability and rounding up to the nearest whole day. If a worker had never been absent due to illness, his or her total duration was considered zero. Finally, we considered a worker to be absent if he or she counted one or more sick leave days, i.e., if his or her total duration was non-zero.

#### Sociodemographics

Companies using Wittyfit provided age, seniority, gender, and job position (manager or employee) of their workers. For the purposes of this study, a manager was defined as an individual who is responsible for overseeing and managing an organization or a group of workers, namely the employees.

### Statistical analysis

Quantitative data (age, seniority, psychosocial factors, duration of sick leave, PWI) were expressed in terms of mean ± SD. Qualitative data (share of women, share of managers, prevalence of the risk of sick leave) were expressed in terms of number of individuals and associated percentage. To construct the SEM of PWI, a two-step approach was performed. Firstly, we fitted several confirmatory factor analysis models to highlight the best structure of psychosocial factors to explain the PWI. Each psychosocial factor has been considered as an item. From a general model, we then made step by step modifications to improve the outcomes. Then, at each step, modification indices were calculated to observe whether the fitted model would improve by adding a path or releasing a constraint. If the change made improved the model, the new model was retained, otherwise a different change was made. Metrics such as the ratio between the Chi-square (CMIN, of χ^2^) and the number of degrees of freedom (df), the Comparative Fit Index (CFI), the Tucker–Lewis Index (TLI), the Akaike’s Information Criteria and Bayesian Information Criteria, and the Root Mean Square Errors of Approximation, were used to compare nested models. If the CMIN/df value is <3.0, it indicates a reasonable fit (Hu and Bentler, 1999). For AIC and BIC criteria, the lower the value, the better the model fit. For the CFI and the TLI, the closer the value is to one, the better (Hu and Bentler, 1999). Regarding the RMSEA, a value close to zero indicated a good fit. A value <0.08 is considered satisfactory, or otherwise poor (Hooper et al., 2008). When no further indices could be calculated, or when the model could no longer be improved, we considered the model to be optimal. Secondly, the optimal SEM was fitted and used to assess the influence of the PWI on sick leave risk. CFA and SEM were performed using the “cfa” and “sem” commands of the “lavaan” package of R, respectively. Once constructed, PWI was computed for each worker. Univariate linear mixed-effect models with company effect as random were fitted to assess the influence of each psychosocial factor on PWI to validate its construction. Finally, a generalized linear mixed-effect model with company effect as random was fitted to assess the influence of PWI on sick leave risk, measured in odds ratio and 95% confidence interval, and a negative binomial generalized linear mixed-effect model with company effect as random was fitted to assess the influence of PWI on sick leave duration, measured in incidence rate ratio and 95% CI. In the absence of data for psychosocial factors, the type of which we have identified as being missing completely at random (Little and Rubin, 2002), hierarchical multiple imputation with company as a cluster was performed using the “mice” command of the eponymous package of R with age, seniority, gender and job position as predictive factors to avoid complete-cases analysis bias (Li et al., 2015; Colin-Chevalier et al., 2022). Multicollinearity of outcomes was checked prior to analysis. Statistical analyses were performed using R (version 4.2.2) (R Core Team, 2021). Unless specified, a *p*-value <0.05 was considered as statistically significant.

## Results

### Participants

**Figure 1:**
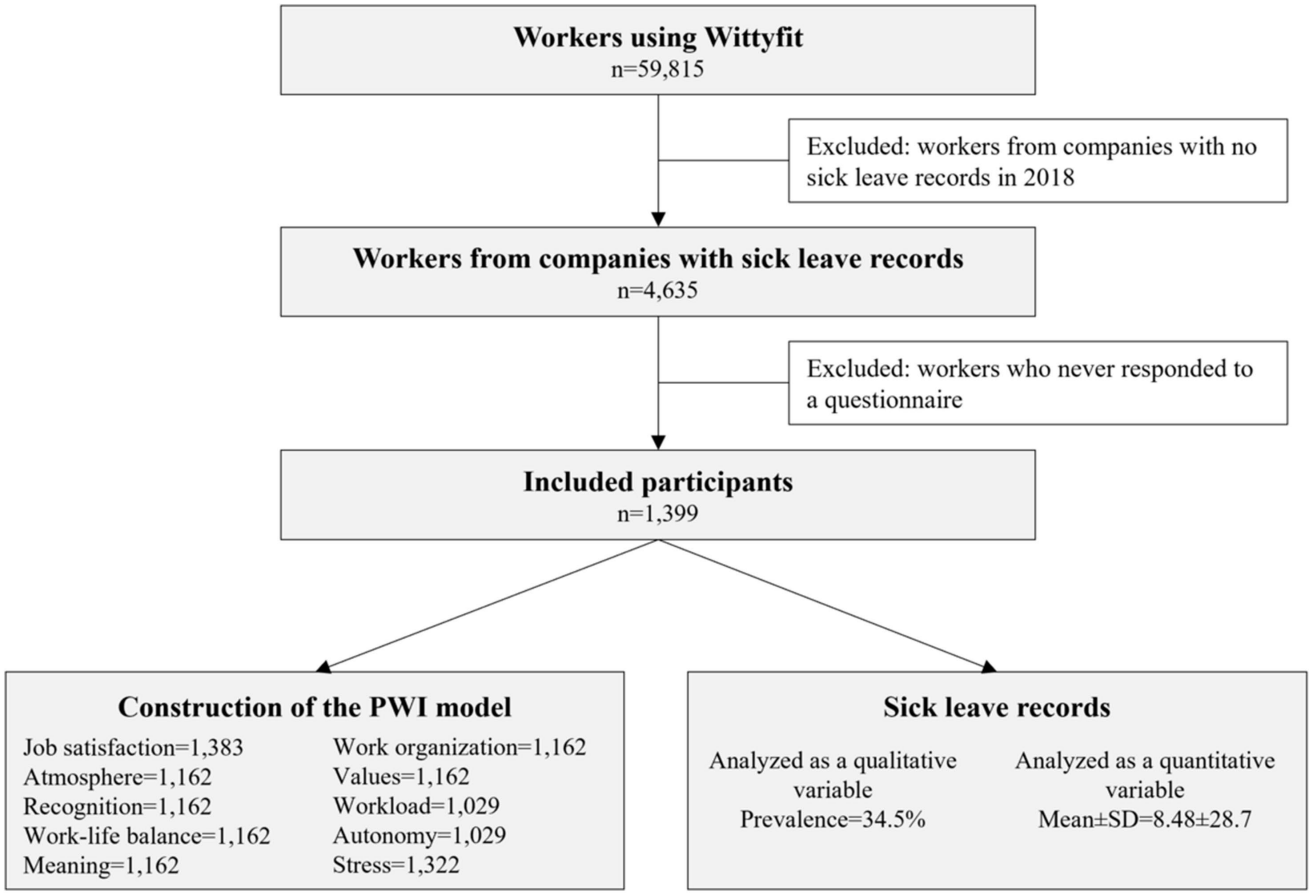
Flowchart of the study. The numbers associated with each psychosocial factor correspond to the number of records (i.e., the number of non-missing values).

Data was collected from 1,399 workers, with a mean age of 39.4 ± 9.4 years, a mean seniority of 9.2 ± 7.7 years, divided into 702 men (50.2%) and 697 (49.8%) women. The workers’ subdivisions consisted of 1,231 employees (88.0%) and 168 being managers (12.0%). Among these workers, 483 (34.5%) were absent due to illness at least once during 2018, and the average duration of sick leave was 8.5 ± 28.7 (estimated kurtosis: 58.9) (Figure 1). Further information on statistics and correlations between psychosocial factors are provided in Supplementary Tables S1, S2.

### Construction of the psychosocial well-being index

**Figure 2:**
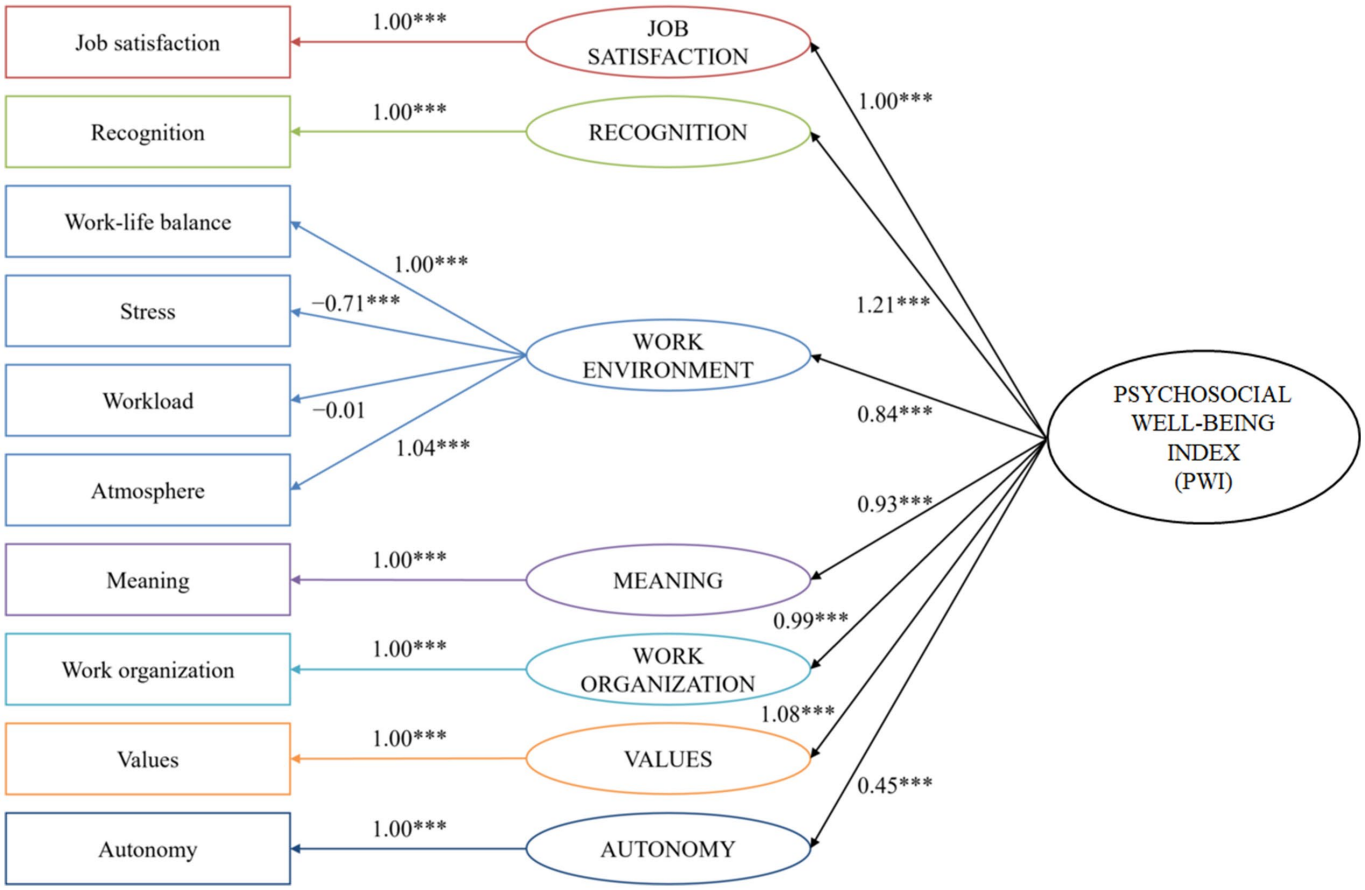
Illustration of the structural equation model of the psychosocial well-being index (simplified version). All measured variables are presented in squares, all latent variables in circles. Covariances between items and variables are not shown but present. ***, *p* < 0.001.

A summary of the model constructed without the effects of covariance between variables is shown in Figure 2.

**Figure 3:**
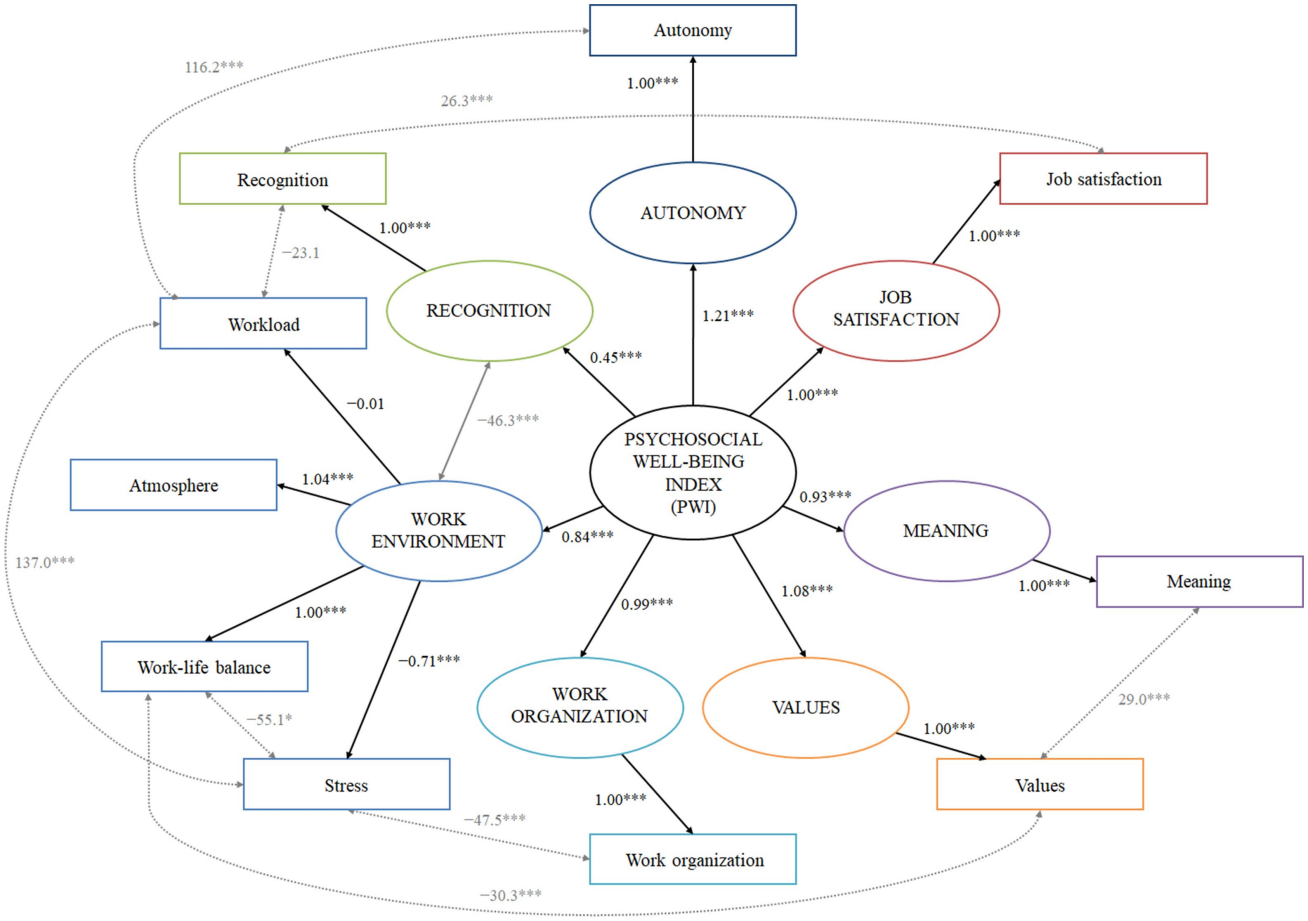
Illustration of the structural equation model of the psychosocial well-being index. All measured variables are presented in squares, all latent variables in circles. Covariances between items and variables are not shown but present. ***, *p* < 0.001.

The complete procedure for creating the psychosocial well-being index was as follows. The first model (model 1) tested was the one in which each psychosocial factor was considered a measure of a distinct latent class, all of which were related to a PWI latent class, with no covariance between items. Except for the latent class related to the workload item (*p* = 0.9), all latent classes showed significant loading on the PWI (*p* < 0.001). This result remained valid in all models. The latent class related to the recognition item was found to have the highest absolute loading on the PWI (*β* = 1.17, 95% CI 1.00–1.34, *p* < 0.001) among all classes. With a RMSEA value of 0.10, the goodness-of-fit was found to be poor. The first change made to the model, leading to model 2, was the addition of the covariance measurement between workload and stress items. This model appeared to be better than model 1 (*χ*^2^-test *p* < 0.001). Despite lower AIC (123,327.6 vs. 123,456.7) and BIC (123,490.1 vs. 123,614.0), goodness-of-fit of the model remained poor (RMSEA = 0.09). In model 3, covariance between workload and autonomy items was added (*p* < 0.001). In addition to being better than the previous model (AIC = 123,214.8, BIC = 123,382.6), the goodness-of-fit of the model appeared to be satisfactory (RMSEA = 0.07) but still improvable. This result remained valid from this model to the optimal and last one. Model 4 was built by grouping two correlated items, work-life balance and stress, into the same latent class (the latent class related to the work-life balance item). The AIC, BIC and RMSEA showed that model 4 was better than model 3. As a result, the new latent variable with two measurement items was retained in subsequent models, and the STRESS class was dropped. Next, model 5 was built by gathering the latent class related to work-life balance and stress and the one related to workload, but its BIC was found to be higher than model 4 (123,339.1 vs. 123,337.6), which led us to test another change. Thus, model 5.2 was built by gathering work-life balance, stress, and workload items in the same latent class. This time, the BIC was found to be lower (123,336.5 vs. 123,337.6) so the model was kept. In the same way, the atmosphere item was added to the latent class which included work-life balance, stress, and workload to build model 6, resulting in a huge increase in goodness-of-fit (RMSEA = 0.06). From this model, the number of latent classes in the models that haven been preserved step-by-step remained stable at seven: six measured by a single item (job satisfaction, recognition, meaning, work organization, values, and autonomy) and the last measured by the four gathered items (work-life balance, stress, workload, and atmosphere). For ease of understanding, each one-item latent class was named by the item that measured it. For example, the latent class measured by the job satisfaction item was called JOB SATISFACTION. In view of the elements that measure it, the four-items latent class was called the WORK ENVIRONMENT class. As model 7, which consisted of adding the recognition item to the WORK ENVIRONMENT latent class was found to be less well-fitting that the model 6 (BIC: 123,382.6 vs. 123,321.4), model 7.2 was built by adding the covariance between stress and work organization items, which fitted better (BIC: 123,299.1). Similarly, model 8 which consisted of adding the recognition item to the largest latent class proved to be less well-adjusted than model 7, so model 8.2 which consisted of adding covariance between the RECOGNITION and WORK ENVIRONMENT latent classes was built and was better adjusted in terms of goodness-of-fit. Finally, models 9–14 were built by adding covariances between meaning and values items, WORK ENVIRONMENT and VALUES latent classes, recognition and workload items followed by work-life balance and stress, job satisfaction and recognition, and lastly work-life balance and values. After model 14, no further improvements could have been made. With a RMSEA of 0.03, model 14 was considered as optimal (Figure 3).

**Figure 4:**
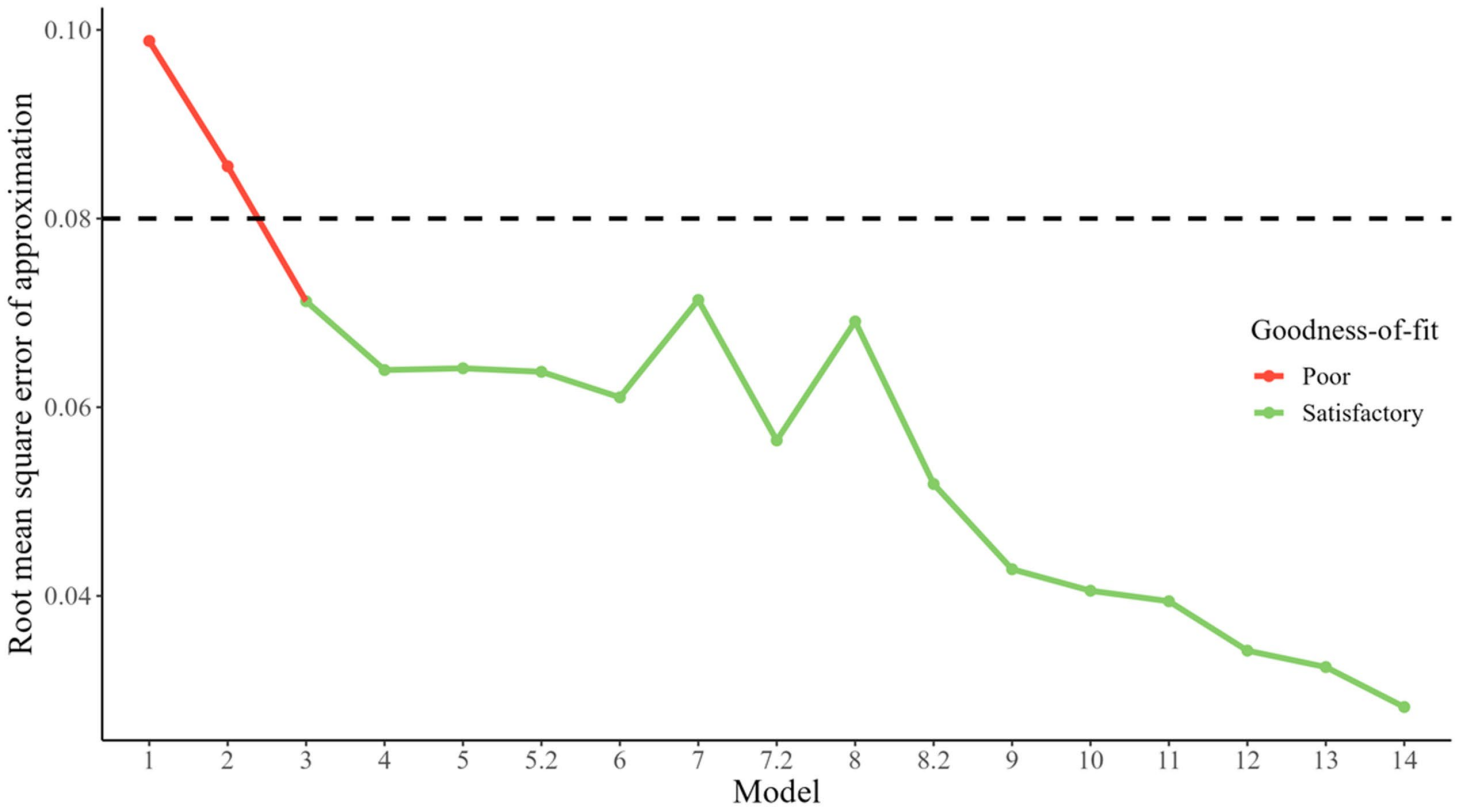
Evolution of goodness-of-fit across models. A point of green color indicates a satisfactory goodness-of-fit of the model (i.e., a RMSEA <0.8), a red color a poor one.

Summary of step-by-step approach to the construction of the optimal model can be found in Figure 4 and Table 1.

**Table 1:** Fit indices of models.

Model	Specifications	CMIN/DF	CFI	TLI	AIC	BIC	RMSEA
Model 1	Each variable as a latent class	14.7	0.93	0.91	123,456.7	123,614.0	0.10
Model 2	Covariance between workload and stress items	11.2	0.95	0.93	123,327.6	123,490.1	0.09
Model 3	Covariance between workload and autonomy items	8.10	0.97	0.95	123,214.8	123,382.6	0.07
Model 4	Work-life balance and stress in the same latent class	6.72	0.97	0.96	123,164.5	123,337.6	0.06
Model 5	Covariance between latent classes 4 (work-life balance/stress) and 8 (workload)	6.75	0.97	0.96	123,160.8	123,339.1	0.06
Model 5.2	Work-life balance, stress, and workload in the same latent class	6.69	0.97	0.96	123,163.5	123,336.5	0.06
Model 6	Work-life balance, stress, workload, and atmosphere in the same latent class	6.21	0.98	0.97	123,148.3	123,321.4	0.06
Model 7	Work-life balance, stress, workload, atmosphere, and recognition in the same latent class	8.13	0.97	0.95	123,209.5	123,382.6	0.07
Model 7.2	Covariance between stress and work organization items	5.46	0.98	0.97	123,120.9	123,299.1	0.06
Model 8	Work-life balance, stress, workload, atmosphere, and recognition in the same latent class	7.68	0.97	0.96	123,189.5	123,367.8	0.07
Model 8.2	Covariance between latent classes 3 (recognition) and (work-life balance/stress/workload/atmosphere)	4.76	0.98	0.98	123,096.4	123,279.9	0.05
Model 9	Covariance between meaning and values items	3.57	0.99	0.98	123,058.9	123,247.7	0.04
Model 10	Covariance between latent classes 4 (work-life balance/stress/workload/atmosphere) and 7 (values)	3.30	0.99	0.99	123,049.9	123,243.9	0.04
Model 11	Covariance between recognition and workload items	3.18	0.99	0.99	123,045.2	123,244.5	0.04
Model 12	Covariance between work-life balance and stress items	2.64	0.99	0.99	123,030.0	123,234.5	0.03
Model 13	Covariance between job satisfaction and recognition items	2.47	0.99	0.99	123,025.3	123,235.1	0.03
Model 14	Covariance between work-life balance and values items	2.11	1.00	0.99	123,016.2	123,231.2	0.03

CMIN, Chi-square; DF, Degrees of Freedom; CFI, Comparative Fit Index; TLI, Tucker-Lewis Index; AIC, Akaike’s Information Criteria; BIC, Bayesian Information Criteria; RMSEA, Root Mean Square Error of Approximation.

### Calculation of the psychosocial well-being index and its influence on sick leave

**Figure 5:**
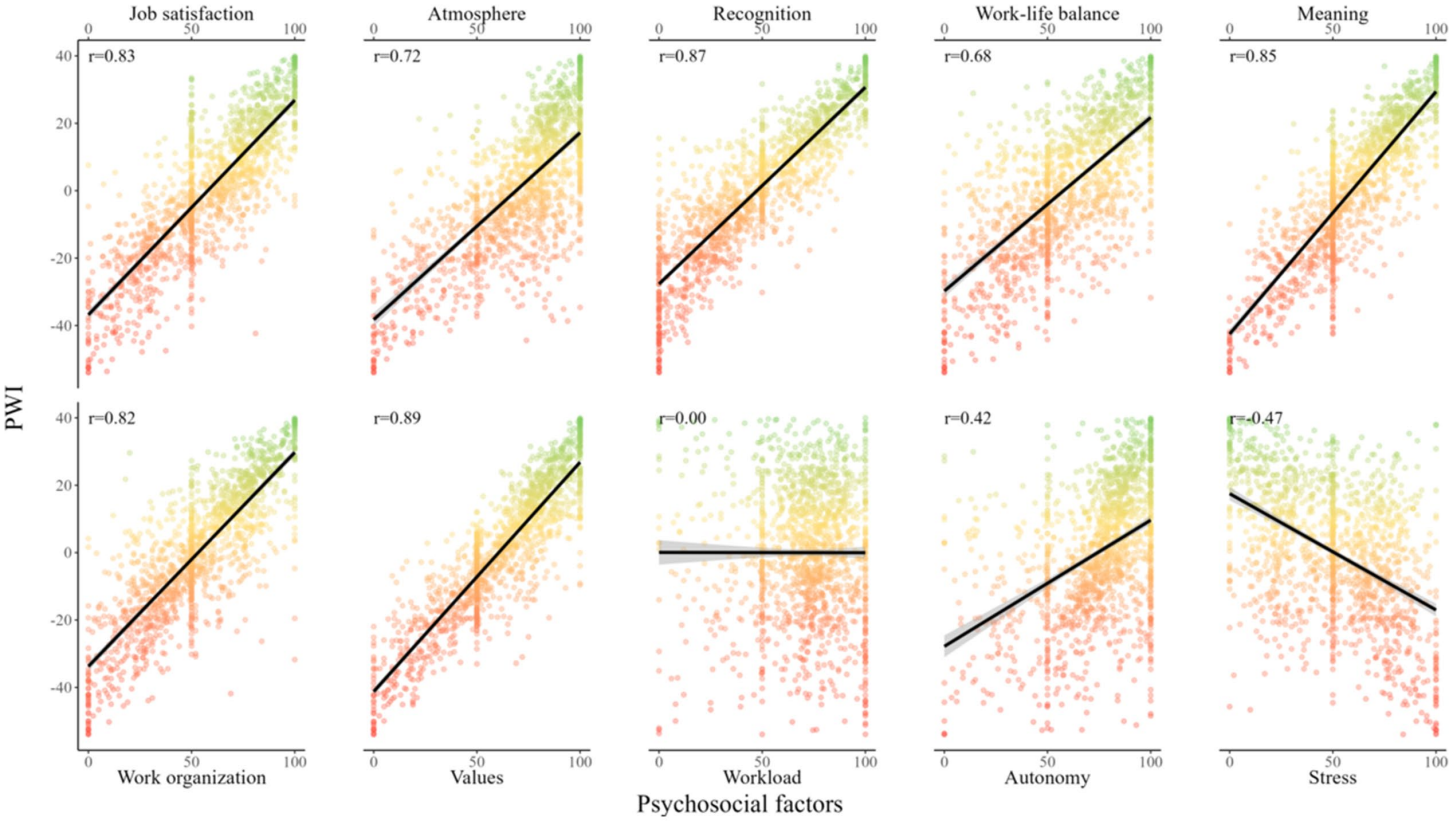
Influence of psychosocial factors on psychosocial well-being index. *r*, Pearson’s correlation between the psychosocial well-being index and the psychosocial factor.

Using the optimal SEM based on data from the same population as that used to fit the model, the PWI of each worker was computed (0.00 ± 19.8, MIN = −53.9, MAX = 40.0). Univariate linear mixed-effect models revealed that, except for workload (0.00, 95% CI −0.05–0.05, *p* = 0.9), all psychosocial factors had an impact on the PWI (*p* < 0.001). In influential order, meaning was the factor with the highest influence across all factors (0.72, 0.69–0.74), followed by values (0.69, 0.67–0.70), job satisfaction (0.64, 0.61–0.66), work organization (0.63, 0.61–0.66), recognition (0.59, 0.57–0.60), atmosphere (0.57, 0.54–0.59), autonomy (0.37, 0.33–0.41) and stress (−0.34, −0.38– −0.31) (Figure 5).

**Figure 6:**
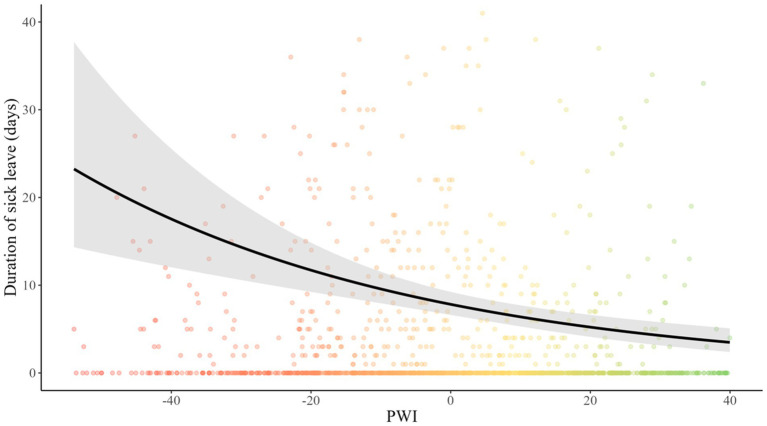
Influence of psychosocial well-being index on duration of sick leave. The y-axis, “duration of sick leave,” is the total duration of sick leave during the year (in days).

Overall, the PWI presents a positive influence on sick leave, and a greater influence than all psychosocial factors. Indeed, for each PWI point, the risk of sick leave was decreased by 2% (OR = 0.98, 95% CI 0.98–0.99, *p* < 0.001). The duration of sick leave also showed a significantly declining trend (IRR = 0.98, 95% CI 0.97–0.99, *p* < 0.001) with an expected duration of 7.8 days of sick leave for an individual with zero PWI. These results remained true with the addition of age, seniority, gender, and job position as confounding variables (Figure 6).

## Discussion

The main results showed that in addition to synthesizing and embracing all considered psychosocial factors, the PWI appeared as a notable factor of sick leave in the workplace. Compared to all other psychosocial factors, PWI demonstrated the greatest influence on sick leave, for both risk and duration.

### Principal results

SEM is a highly effective multivariate technique that is increasingly used in scientific research to evaluate complex causal relationships among variables. Unlike other modelling approaches, SEMs allow us to measure supposed variables that could not be measured previously, also called latent variables. This feature offers researchers the opportunity to create measurement scales, which is particularly useful in the health sector (Yazdanirad et al., 2020; Maier et al., 2021). In this study, we aimed to create an index able to measure workers’ psychosocial well-being (or quality of life), called PWI. A total of 10 psychosocial factors were selected, combined, and weighted in a SEM to allow identification and calculation of a conceptualized index. Except for workload (even if covariances between workload and recognition, autonomy, and stress are computed in the model), all psychosocial factors were found to be significantly related to psychosocial well-being. This result suggests that, as desired, PWI provides a holistic understanding of worker health. In their desire to promote health at work, organizations should strive to work on all dimensions of psychosocial well-being since each contributes to its improvement. Following the construction of the PWI, we analyzed how psychosocial well-being could increase the risk of sick leave in workers. Our findings suggest that workers who may experience discomfort due to their working conditions are at greater risk of deteriorating health, resulting in an increased risk of sick leave. In addition to reducing the risk of absence due to illness, we found that workers’ average duration of sick leave over the year was lower as their PWI increased, and vice versa. In view of all these results, it appears that the improvement of psychosocial working conditions could thus contribute significantly to reducing absenteeism due to illness (Mathisen et al., 2022). In theory, interventions to promote psychosocial well-being in the workplace should lead to a decrease in sick leave in the workplace, in addition to, and most importantly, preserving workers’ health.

### Limitations

We acknowledged some limitations within the study. The possibility of self-report bias and affective bias due to social desirability may exist in workers’ self-reported responses to surveys. However, Wittyfit minimizes bias by anonymizing all user data, which enables users to express themselves freely (Dutheil et al., 2017). The study population was also limited to French workers, although differences in sick leave risk exist between countries. However, the prevalence of sick leave in our study, measured at 34.5%, is consistent with the literature (Antczak and Miszczyńska, 2021), which supports our results. Also, the limited data sample used in the study may not be a true representation of the entire population of French workers, notably in terms of companies, where differences in the prevalence of sick leave may exist. To reduce this bias, all models were transformed into mixed models in which the firm effect was considered as a cluster. As a cross-sectional study, causality cannot be established. It is possible that workers who have better psychosocial well-being are less likely to take sick leave (Hystad et al., 2011; North et al., 1996), but it is also possible that taking sick leave affects psychosocial well-being and intentions to go back to work (Hedlund et al., 2021). No information underlying the illnesses that caused workers to stop working was provided by the client companies included in the study. However, it is known that shorter absences tend to be caused by illnesses with short recovery time (cold, cough, and influenza), and longer absences by illnesses with long recovery time, mainly associated with mental health issues (Demou et al., 2018). Nevertheless, even low-recovery-time illnesses such as cold (Bagwath Persad et al., 2017) or high-recovery-time illnesses as depression (Bonde, 2008), can be caused by psychosocial factors. Future studies that examine the reasons for sick leave could strengthen the extent of the association between psychosocial well-being and sick leave.

### Comparison with prior work

The holistic aspect of PWI reinforces the idea that understanding worker well-being cannot be done without a comprehensive approach, which is the stated goal of the Wittyfit platform (Dutheil et al., 2017). Among the factors included in the model, meaning proved to be the most relevant in explaining a worker’s psychosocial well-being, which suggests that meaning at work might be the main driver of workers’ well-being. It is not surprising since this concept is known to offer an existential justification for work and gives meaning to life (Lee, 2015), which goes beyond professional life, as well as increasing performance (Wingerden and Van der Stoep, 2018). Slightly behind meaning, values and job satisfaction emerged as factors for improving workers well-being as previously observed (Kantek and Kaya, 2017). The lack of effect of workload may be surprising when it is known that it can predict mental strain, but mainly when combined with job control (e.g., autonomy) (Karasek, 1979; Karasek et al., 1981; Theorell et al., 1990). In fact, while workload alone can have a significant influence on psychosocial well-being, its effects are often linked to other factors such as work-related stress, which may explain the lack of influence (Adams and Nino, 2024). Although workload was not directly linked to psychosocial well-being in the model, the significant covariance effects found with other variables, and its attachment to the latent class also defined by atmosphere, work-life balance and stress, factors known to influence each other (Theorell et al., 1990; Buruck et al., 2020), reinforced our decision to retain this parameter. The association between psychosocial well-being and sick leave is well-known (Mathisen et al., 2022; Hedlund et al., 2021). Our results confirm the role of psychosocial factors in the risk of sick leave. For example, a study in the Norwegian armed forces found that psychosocial resilience could predict the likelihood of absence due to illness (Hystad et al., 2011). More generally, a study in pregnant employees found that poor health was associated with more sick leave (Pedersen et al., 2021). Similarly, we found that psychosocial well-being could reduce the average length of sick leave for workers. A study in a Dutch telecommunications company showed that coping, which refers to how individuals deal with stressors, is an important factor in promoting psychosocial well-being and reducing the duration of sickness absence (van Rhenen et al., 2008). The duration of sickness absenteeism has also been observed to increase in response to adverse psychosocial job conditions (Leach et al., 2022).

## Conclusion

This study highlighted the importance of psychosocial well-being, but most importantly of the PWI, and its association with sick leave in the workplace. The findings suggest that interventions aimed at improving psychosocial well-being factors could help reduce sick leave risk and mean duration. This study provides valuable insights for employers and policymakers to implement measures to improve workers’ psychosocial well-being, which could lead to a healthier and more productive workforce. Further research could investigate the effectiveness of interventions to improve psychosocial well-being in reducing sick leave and improving overall health outcomes.

## Data Availability

The datasets presented in this article are not readily available because data cannot be transmitted without the prior consent of the company’s corporate clients, except to the University Hospital of Clermont-Ferrand, France, which may use the data for research purposes. Requests to access the datasets should be directed to rcolin@cegid.com.
