# Supplementary material for: Psychosocial well-being index and sick leave in the workplace: a structural equation modeling of Wittyfit data
Source: Front Psychol. 2025 Jan 24;16:1385708. doi: 10.3389/fpsyg.2025.1385708 (PMC11802550; doi:10.3389/fpsyg.2025.1385708)
Supplement: Supplementary file 1 [file Table_1.docx]

**Table S1**. Statistics on Psychosocial Factors.

| **Variable** | **n** | **MIN** | **MAX** | **Mean±SD** | **Shapiro-Wilk test (statistic and *P*-value)** |
| --- | --- | --- | --- | --- | --- |
| Job satisfaction | 1399 | 0 | 100 | 57.8±25.7 | 0.97*** |
| Atmosphere | 1399 | 0 | 100 | 69.0±25.9 | 0.91*** |
| Recognition | 1399 | 0 | 100 | 47.4±29.5 | 0.96*** |
| Work-life balance | 1399 | 0 | 100 | 57.8±26.3 | 0.97*** |
| Meaning | 1399 | 0 | 100 | 59.1±23.4 | 0.97*** |
| Work organization | 1399 | 0 | 100 | 53.2±25.7 | 0.98*** |
| Values | 1399 | 0 | 100 | 60.6±26.0 | 0.96*** |
| Workload | 1399 | 0 | 100 | 70.9±21.2 | 0.93*** |
| Autonomy | 1399 | 0 | 100 | 74.3±22.5 | 0.90*** |
| Stress | 1399 | 0 | 100 | 50.7±26.9 | 0.97*** |

Legend: ‘n’: number of individuals, ‘MIN’: minimum value, ‘MAX’: maximum value, ‘SD’: standard deviation ‘***’: *P*<.001.
