# Supplementary material for: Psychosocial well-being index and sick leave in the workplace: a structural equation modeling of Wittyfit data
Source: Front Psychol. 2025 Jan 24;16:1385708. doi: 10.3389/fpsyg.2025.1385708 (PMC11802550; doi:10.3389/fpsyg.2025.1385708)
Supplement: Supplementary file 2 [file Table_2.docx]

**Table S2**. Matrix of Pearson’s Correlation Coefficients between Psychosocial Factors.

| **Variable** | **1** | **2** | **3** | **4** | **5** | **6** | **7** | **8** | **9** | **10** |
| --- | --- | --- | --- | --- | --- | --- | --- | --- | --- | --- |
| **1**. Job satisfaction | -- |  |  |  |  |  |  |  |  |  |
| **2**. Atmosphere | 0.57*** | -- |  |  |  |  |  |  |  |  |
| **3**. Recognition | 0.70*** | 0.53*** | -- |  |  |  |  |  |  |  |
| **4**. Work-life balance | 0.52*** | 0.51*** | 0.49*** | -- |  |  |  |  |  |  |
| **5**. Meaning | 0.65*** | 0.56*** | 0.69*** | 0.54*** | -- |  |  |  |  |  |
| **6**. Work organization | 0.62*** | 0.54*** | 0.67*** | 0.54*** | 0.66*** | -- |  |  |  |  |
| **7**. Values | 0.69*** | 0.58*** | 0.72*** | 0.50*** | 0.75*** | 0.68*** | -- |  |  |  |
| **8**. Workload | −0.03 | 0.03 | −0.04 | −0.06* | 0.03 | −0.02 | 0.02 | -- |  |  |
| **9**. Autonomy | 0.34*** | 0.30*** | 0.33*** | 0.24*** | 0.35*** | 0.33*** | 0.36*** | 0.26*** | -- |  |
| **10**. Stress | −0.40*** | −0.35*** | −0.33*** | −0.43*** | −0.35*** | −0.44*** | −0.36*** | 0.27*** | −0.14*** | -- |

Legend: ‘*’: *P*<.05, ‘***’: *P*<.001.
